# Effects of Glucan and Vitamin D Supplementation on Obesity and Lipid Metabolism in Diabetic Retinopathy

**DOI:** 10.2174/1874091X01812010036

**Published:** 2018-03-30

**Authors:** Martina Závorková, Vaclav Vetvicka, Josef Richter, Vlastimil Kral, Ivana Liehnova, Dobiasova L. Rajnohova

**Affiliations:** 1 Official Clinic of UJEP Masaryk Hospital, Regional Health, Usti and Labem, Czech Republic; 2 University of Louisville, Department of Pathology, Louisville, KY, USA; 3 Health Institute with headquarters in Usti and Labem, Usti and Labem, Czech Republic

**Keywords:** Glucan, Vitamin D, Diabetes, Obesity, Diabetic retinopathy, Supplementation, Clinical trials

## Abstract

**Background:**

Diabetes mellitus is a chronic disease manifested by an increase of blood glucose.

**Objective:**

To evaluate the effects of glucan and vitamin D supplementation in patients with diabetic retinopathy.

**Method:**

We evaluated the effects of 3-month supplementation with glucan and vitamin D in 54 patients with diabetic retinopathy. We measured levels of vitamin D, cholesterol, HDL cholesterol, and triglycerides.

**Results:**

The supplementation strongly decreased the cholesterol levels and improved the levels of HDL cholesterol. In addition, vitamin D levels were strongly improved, but still not at optimal values.

**Conclusion:**

From our data, we concluded that glucan and vitamin D supplementation strongly influence lipid metabolism and have positive effects on human health.

## 1 INTRODUCTION

Diabetes mellitus (DM) is a chronic, lifelong disease manifested by an increase in blood glucose levels. The dysregulation of glucose results from a decrease of insulin production or from systemic resistance to insulin effects. Currently, approximately 10% of the population of developed countries suffers from DM and this trend is increasing. About 20% of patients with DM suffer from blood vessel damage, causing significant stress on healthcare and resulting in an urgent need of new, wide-ranging preventive actions to lower DM-related risks. These actions include prevention of lipid metabolism, prevention of obesity, addressing high blood pressure and inflammation, smoking, low physical activity, and other problems. This report follows up on previous studies evaluating the effects of glucan and vitamin D on apolipoproteins in diabetic retinopathy (DR). In this study, we focused on levels of vitamin D, nutrition and metabolism of lipids, particularly levels of cholesterol, triglycerides, and cholesterol: HDL cholesterol ratio.

Lately, commercial treatment of DR is often accompanied by the addition of nutritional supplements aiming to use antioxidants such as carotenoids, lutein, or zeaxanthin as prevention [1]. Recently, material isolated from broccoli (sulforaphane) was used not only due to its anti-cancer properties but also for influencing vascular complications in diabetes and for reduction of liver glucose [2, 3]. Actual epidemiological studies suggest that the duration of DM, and elevated blood pressure and glycemic levels have significant effects on the development of DR. Body mass index (BMI) is the most commonly used indicator of nutrition state. Individuals with high BMI have a high risk of development of DR. In overweight people, this risk is less pronounced [4]. In cases of BMI higher than 40, the risk of developing DR is five times higher than in people with normal weight [5].

Higher levels of cholesterol and lipids are basic parts of metabolic syndrome. They clearly result in higher risk of vision damage and DR. HDL and triglycerides levels play an unknown role; the clinical findings are statistically insignificant. On the other hand, higher physical activity is one of the preventive steps leading to significant reduction of DR development [5]. Polysaccharides and glucans, in particular, play a significant role in the prevention of the metabolic syndrome and reduction of obesity [6-8].

Vitamin D refers to a group of fat-soluble secosteroids responsible for increasing intestinal absorption of calcium, magnesium, phosphate, and zinc. It is a prohormone and, together with calcitonin and parathormone, is the main biological regulator of calcium and phosphate metabolism. Adequate levels of vitamin D are the basic requirements for optimal bone mineralization and photosynthesis of vitamin D3 in the epidermis. In addition, vitamin D receptor in keratinocytes changes these cells into a unique photoendocrine system stimulated by ultraviolet B. The binding of vitamin D to vitamin D receptor increases production of cathelicidins, which have significant microbicidal activity and form an important part of the nonspecific immune system [9]. Clearly, vitamin D plays an important role in defense reactions, which is shown in the reduction of cancer, hypertension, and vascular system disease. The combination of factors, such as lifestyle, nutrition, anthropometry, sociodemographic, and genetic polymorphism, reflects quality and validity of life [9]. More information about the protective role of vitamin D in DM II risk is becoming available. This information formed the basis for our study of the possible effects of vitamin D levels in patients with DR and of the possible role of vitamin D supplementation.

New findings on transporters, receptors, and enzymes connected to cholesterol metabolism helped us to understand the mechanisms of sterol absorption [10]. Cholesterol and phytosterol transport into enterocytes is a tightly regulated process. Any disruption of this balance can aid in the development of hypercholesterolemia. Some parts of nutrition, particularly the ones able to change cholesterol absorption are involved in keeping the optimal balance. Phytosterols, phospholipids, soluble fibers, stearic acid, and other food components can serve as an example [10]. Unbalanced intake of these components varies by country. Compared to Asia and Africa, the European situation is worse, making the possibility of food supplementation by purified components more attractive.

Glucans and their actions have long been of interest to our research group [11-15]. Glucans belong to a group of physiologically active compounds, sometimes called biological response modifiers, and represent highly conserved structural components of cell walls in yeast, fungi, and seaweed. The term glucan is sometimes used as a chemical name of glucose polymer and represents a group of chemically heterogeneous carbohydrates consisting of various numbers of glucose molecules bound together in several types of linkages. The role of glucan as a biologically active immunomodulator has been well established for over 50 years.

Despite numerous international agreements on fibers classification, there is clear proof showing the significance of dietary fibers in obesity and metabolic syndrome. Glucan, which can be classified as a fiber, has various functional and bioactive properties. Readers seeking the details regarding the biological action of glucan should see a recent monography [14]. Its positive effects on insulin resistance, dyslipidemia and hypertension have also been reported [16]. Our group has found numerous positive effects of glucan both in adult and children populations. Reduction of cholesterol levels by the fiber is a focus of constant worldwide attention and often results in additional activities evaluating not only the quality of material and its purity but also glucan characteristics based on the source [17-19].

In this report, we focused on the evaluation of levels of vitamin D, nutritional state, and lipid metabolism, particularly cholesterol, HDL, triglycerides, and cholesterol/HDL ratio. We used DM disease not only for a close relationship between obesity and DM but also for the relationship with adipokines and most of all leptin. In addition, glucan has a significant role in stress reduction [20, 21]. The actions of glucan and vitamin D led us to investigate the possible action of supplementation with these molecules on patients with DR.

## 2 MATERIALS AND METHODOLOGY

### 2.1 Glucan

Yeast-derived insoluble Glucan #300 (>85% dry w/w basis) was purchased from Transfer Point (Columbia, SC, USA). This glucan contains 96% carbohydrates and 2.1% proteins. Neutral sugar analysis confirmed 91.3% glucose and 8% mannose. Glucan was taken on an empty stomach, followed by 100 ml of water and 30-minute rest prior to any food intake.

### 2.2 Vitamin D

Vitamin D (cholecalciferol, D3, Vigantol) was manufactured by Merck (Darmstadt, Germany). One ml of solution contains 20,000 IU of vitamin D3, one drop contains 500 IU. All patients were instructed to ingest vitamin D with fat-containing food.

### 2.3 Protocol

We explained the experimental protocol and obtained consent forms from all participating patients. This study was Institutional Review Board approved and performed in full agreement with the Helsinki declaration (revised version 2000.09.01), and in full compliance with the Czech Republic’s clinical testing rules.

We reviewed and divided 54 patients into four groups: Group A consisted of patients supplemented with beta-glucan and vitamin D; Group B consisted of patients supplemented with vitamin D and placebo; Group C consisted of patients supplemented with vitamin D only, and Group D represented patients getting no supplementation. Individual groups of DR patients were supplemented for 3 months (one dose per day), in addition to normal treatment. The glucan dose was 500 mg/day, vitamin D dose was based on age, weight and season; the placebo consisted of pills in the same design, shape, and color. The tests were performed at the beginning and the end of the experiment.

All the patients were established by skin phototype, weight, height, BMI, a body shape index (ABSI) [22-24], and starting levels of vitamin D and lipid metabolism. Cholesterol, HDL cholesterol, and triglycerides were evaluated using standard methods used in our clinical department. Each patient in our study obtained the same type of medication throughout the entire duration of the study.

### 2.4 Tests 

Vitamin D levels were measured by an ELISA assay using standards recommended by the manufacturer (DRG Instruments, Germany). Based on the manufacturer’s information, average values for a healthy common 58-year-old Caucasian population are 26.1 ng/ml in males and 30.2 ng/ml in females. A vitamin D deficit is considered when levels are below 10 ng/ml, insufficient levels range between 10–29 ng/ml, and normal levels range between 30–100 ng/ml. Samples were taken in the morning on the empty stomach. Anthropometric evaluations were part of the initial medical examinations. Weight and height were measured, and BMI levels were calculated.

### 2.5 Statistical Analysis

Paired-test statistical significance was evaluated (GraphPad Prism 5.04; GraphPad Software, USA). An average and standard deviation were evaluated after determining standard value composition (D’Agostino, Pearson). In case of nonstandard composition, values were converted into logarithms.

## 3 RESULT

Table **1** summarizes basic characteristics of our patients. Average age (above 60 years of age) was the same in all groups. Differences in BMI are not statistically significant between individual groups or between the beginning and end of the study. Table **2** shows frequency of BMI values according to the National Institutes of Health classification and WHO suggestions. It is clear that only 10.4% population showed normal weight; we found increased weight in 27.3% of women and 38.5% of men (*P*=0.352). Obesity of the first degree was found in 29.2% of the entire group of patients, obesity of the second degree in 18.8%, with highest being in the women group (27.3%). Extreme obesity (obesity of the third degree) was found in 8.3% with no difference between men and women. Differences between ABSI values were not significant, most probably due to the high spread of individual values. When comparing BMI and new BMI, we found this ratio to be statistically highly significant with linear regression nBMI/ABSI (*P*=0.0004). Skin phototypes did not differ among our groups.

Fig. (**1**) summarizes the levels of cholesterol. We found a strong decrease in cholesterol level from 4.92±1.14 mmol/l to 4.69±1.41 (*P*=0.03) in Group A, a decrease from 5.0±1.52 to 4.57±1.19 in Group B was also significant (*P*=0.045). Changes in Group C were not significant (4.79±1.09 to 4.77±1.21, *P*=0.618). The control group had average levels of 6.69±1.95, which were statistically different from Groups A, B, and C.

Fig. (**2**) shows changes in HDL levels with significant changes from 1.3±0.43 to 1.6±0.48 (*P*=0.044) in Group A. The differences in all other groups were not significant. Fig. (**3**) summarizes the ratio of cholesterol/HDL with no statistically significant changes in any tested group. Next, we focused on possible changes in levels of triglycerides Fig. (**4**). Again, no statistically significant changes in any tested group were found.

In Fig. (**5**), we summarized levels of vitamin D at the beginning of our study. Almost 30% of tested individuals have levels of vitamin D lower than 15 ng/ml; the one patient with levels over 30 ng/ml was quite exceptional. Levels of vitamin D after 3-month supplementation are shown in Fig. (**5**).

Our study found that even 3 months of supplementation with individual doses (between 400 and 8000 IU) did not help the patients with diabetes to reach normal values. However, all groups demonstrated increase, which was significantly seen in Groups A and B, In addition, we found a significant relationship between BMI values and levels of vitamin D. The effects of vitamin D supplementation were the same in both women and men groups. The increase in the men group was from 15.23 ng/ml to 23.88 ng/ml; whereas, in the women group, it was from 13.1 ng/ml to 22.73 ng/ml. When we measured the weight, we found a statistically significant difference in vitamin D values in relation to the BMI values. Groups with higher weight and obesity of the first degree had levels of vitamin D averaging 15.5 ng/ml; whereas, patients with BMI over 35 kg/m2 had levels averaging 12.75 ng/ml.

## 4 DISCUSSION

Our findings of the weight distribution in patients with DM are in agreement with data in the literature [4, 25]. The above average weight in these groups most probably reflects inadequate physical activity and suboptimal nutrition. When compared with the average population in the Czech Republic of the same age, the differences in weight are statistically significant. In sampling 6,737 people from different parts of the Czech Republic, the BMI levels in males were 28.1±0.225 kg/m2 and in females were 26.9±0.47 kg/m2. Obesity (BMI over 30) was found in 29% of males and 25% of females [26-28]. Zhou speculates that obesity is not a fully unequivocal criterion for DR induction and stresses the need for additional studies [4]. A relationship between obesity and vitamin D deficit has been repeatedly studied, with results suggesting the need of supplementing DR patients with vitamin D.

Glucans represent both insoluble and soluble viscous types of fiber. Chemically, they are heterogeneous non-starch polysaccharides, which form structural compounds of the cell wall of some microorganisms including yeast and algae, and even protists, mushrooms, and grain. It is generally accepted that dietary fiber (including β-glucans) of various origin have positive health effects. Numerous experimental animal studies and clinical human tests provide evidence that dietary fiber intake not only supports health generally but also reduces the risk of onset and development of the most contemporary widespread noncommunicable diseases such as cardiovascular disease, cancer, and diabetes type 2 [29]. The hypocholesterolemic effects of glucan are well documented [30, 31], but still not fully understood. It is attributed to the ability of soluble DF to form viscous solutions that prolong gastric emptying and inhibition of the transport of triglycerides and cholesterol across the intestine and to reduce total LDL-lipoprotein concentration [32]. Consequences of an increased viscosity of the luminal contents will appear in the amplification of the thickness of the water layer and in the decrease of uptake of cholesterol from the intestine lumen. Dietary fiber fractions from mushrooms were found to modulate cholesterol-related gene expression [33]. Lim [34] used a hamster experimental model to apply β-glucans (“polycan”) from yeast-like fungus *Aureobasidium pullulans*. The authors concluded that polycan decreased the atherosclerotic effects, hyperlipemia, and hepatic damage of liver, which were induced by means of high-fat diet. An additional study found that β-glucan treatment reduced the inflammation induced by a high cholesterol diet [35]. In a rat experimental model, β-glucan reduced total cholesterol, triglyceride, and malondialdehyde levels up to 42% [36]. In men, long-term clinical studies using soluble forms of β-glucans demonstrated decreased blood cholesterol in hypercholesterolemic patients [37-39].

In our collection, we found a very limited outdoor physical activity, resulting in minimal exposure to the sun [9]. However, the limited movement resulting from additional DR-related diseases is not the only reason for the low exposure. Additional reasons include critically low sunshine in the region (only 800 hr/year) and further reduction of ultraviolet B due to the ozone layer or and chemical pollution. The relation between BMI and vitamin D is clear and our findings also showed an increased risk of inflammation. Lately, significant attention has been focused on relations between obesity and vitamin D. Several studies described relations between changes in vitamin D levels and changes in BMI. Vitamin D supplementation did cause not only a decrease of BMI but also a decrease of inflammatory markers. In addition, this observation was found not only in people with diabetes but also in a healthy population [40]. Other studies suggested protective effects of vitamin D in the development of diabetes Type II [25].

In addition, vitamin D deficiency has been implicated in the pathophysiology of various inflammatory diseases such as Crohn’s disease and rheumatoid arthritis. It is documented that supplementation with vitamin D lowers the risk of developing DM, which led to recommendations of vitamin D supplementation as DM prevention. Vitamin D significantly affects glucose metabolism, regulates exocytosis of insulin, directly affects stimulation of insulin receptors, improves intake of glucose in peripheral cells and regulates insulin resistance.

## CONCLUSION

Our findings led us to supplement our patients also with glucan, aiming to potentially influence both their obesity and metabolic syndrome [16]. Our findings show that the addition of glucan significantly influenced the lipid metabolism. Clearly, the supplementation of food with glucan has a strong potential in preventive medicine [1, 10, 17-19, 28]. The ongoing increase in DM occurrence represents a significant problem and a strong need for further studies and implementation of preventive actions. To succeed, we need to understand the etiology of this disease in details. Evaluation of many risk factors is important [5]. From our results, we can conclude that supplementation with glucan and vitamin D resulted in significant increase of vitamin D levels, improvements of HDL levels, and strong decrease of the total level of cholesterol, supporting the hypothesis that glucan and vitamin D supplementation has positive effects on human health.

## Figures and Tables

**Fig. (1):**
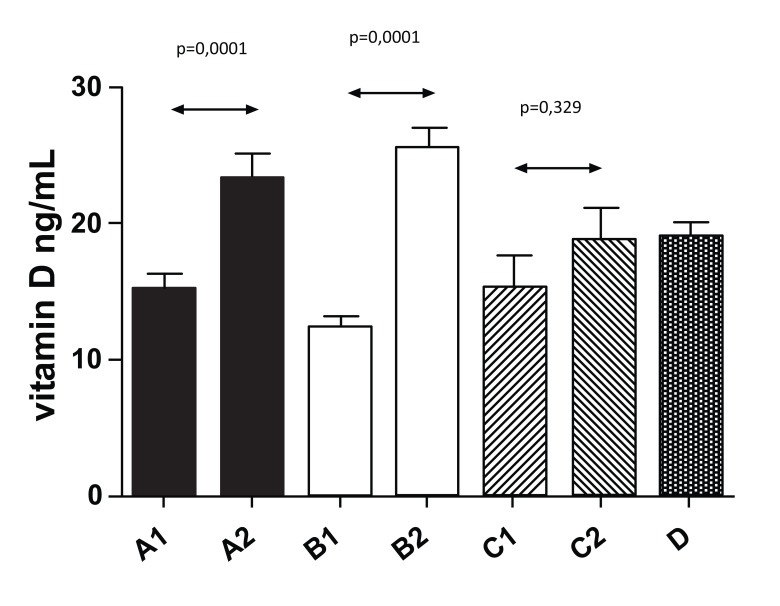
Effect of glucan and vitamin D supplementation on cholesterol levels. Description of individual groups is given in Material and Methods. 1 means data obtained at the beginning of the study, 2 means data obtained at the end of the study.

**Fig. (2):**
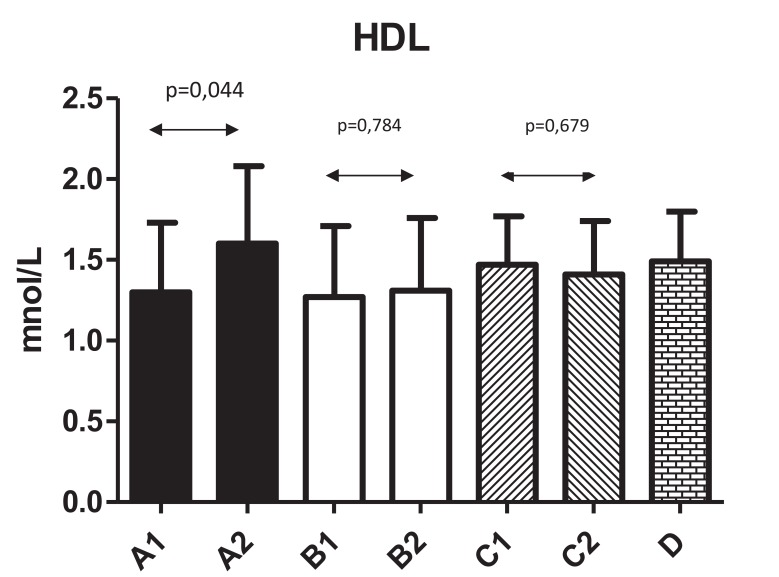
Effect of glucan and vitamin D supplementation on HDL cholesterol levels. Description of individual groups is given in Material and Methods. 1 means data obtained at the beginning of the study, 2 means data obtained at the end of the study.

**Fig. (3):**
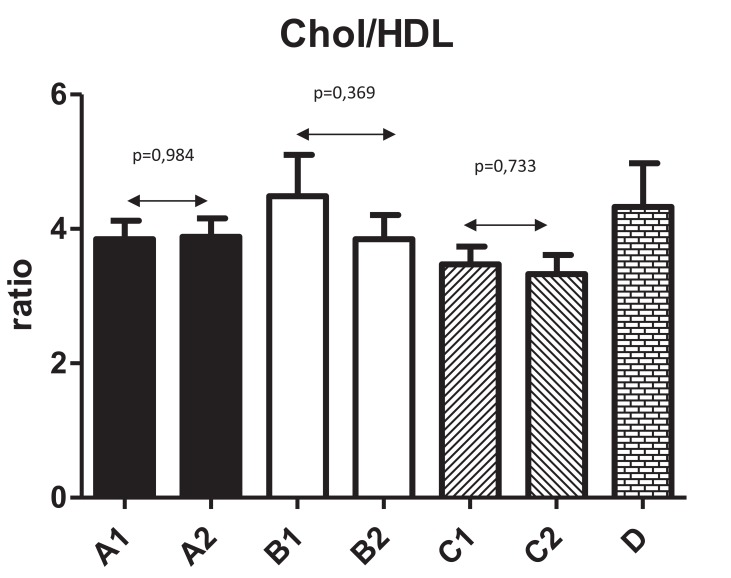
Effect of glucan and vitamin D supplementation on ratio cholesterol/HDL levels. Description of individual groups is given in Material and Methods. 1 means data obtained at the beginning of the study, 2 means data obtained at the end of the study.

**Fig. (4):**
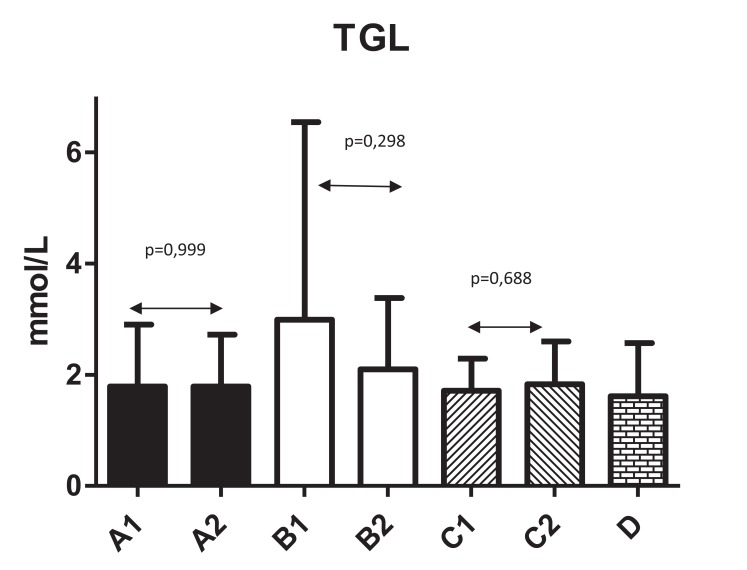
Effect of glucan and vitamin D supplementation on triglyceride levels. Description of individual groups is given in Material and Methods. 1 means data obtained at the beginning of the study, 2 means data obtained at the end of the study.

**Fig. (5):**
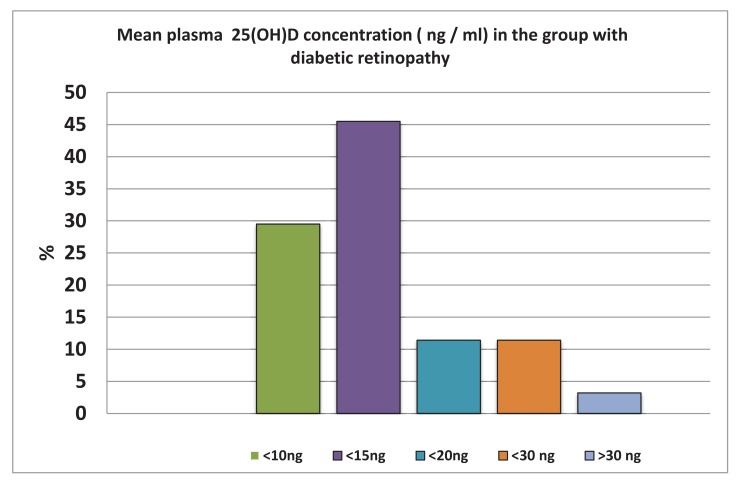
Prevalence of ear mean plasma 25(OH) D concentration (ng/ ml) in the group with diabetic retinopathy. 1 means data obtained at the beginning of the study, 2 means data obtained at the end of the study.

**Table 1:** Basic characteristics of patients.

Characteristics	Group A	**Group B**	**Group C**	**Group D**
**n ( female / male )**	21	21	12	14
**age (yr)**	66,6	66,7	67,03	63,7
**BMI**	32,6	30	31,1	31,78
**new BMI**	31,32	31,1	31,85	31,05
**ABSI**	0,087	0,092	0,087	0,088
**phototype**	2,9	2,7	2,6	3
**vitamin D**	15,23	12,8	17,81	23,3
**total cholesterol mmol/L**	4,59	5,01	4,79	6,43
**HDL cholesterol mmol/L**	1,27	1,28	1,41	1,49
**triglyceride mml/L**	1,79	2,49	1,71	1,8
**cholesterol/HDL ratio**	3,875	3,48	3,47	4,33

**Table 2:** Frequency of BMI values according to the National Institutes of Health classification and WHO suggestions.

	**BMI ( kg/m2 )**		**female %**	**male %**	**group**
**underweight**	< 18,5		0	0	0
**normal weight**	18,5	- 24,9	13,5	7,7	10,4
**overweight**	25,0 - 29,9		27,3	38,5	33,3
**obesity I**	30,0	- 34,9	22,8	34,6	29,2
**obesity II**	35 - 39,9		27,3	11,5	18,8
**Extreme obesity**	40,0	+	9,1	7,7	8,3

## LIST OF ABBREVIATIONS

ABSI: = A Body Shape Index
BMI: = Body Mass Index
DM: = Diabetes Mellitus
DR: = Diabetic Retinopathy
